# Classical and Bayesian predictions applied to *Bacillus* toxin production

**DOI:** 10.1007/s13205-016-0527-2

**Published:** 2016-09-26

**Authors:** Karim Ennouri, Rayda Ben Ayed, Maura Mazzarello, Ennio Ottaviani, Fathi Hertelli, Hichem Azzouz

**Affiliations:** 1 Centre of Biotechnology of Sfax, 3038 Sfax, Tunisia; 2 OnAir s.r.l.-26, Via Carlo Barabino, Genoa, Italy

**Keywords:** *Bacillus thuringiensis*, Delta-endotoxins, Hadamard matrices, Bayesian method, Mean square error, Least square method

## Abstract

*Bacillus thuringiensis* is a bacterium with unusual properties that make it useful for pest control in ecoagriculture. It can form a parasporal crystal containing polypeptides (also called delta-endotoxins). These entomopathogenic toxins are made during the stationary phase of the bacterial growth cycle and were initially characterized as an insect pathogen. Nowadays, the use of saturated two-level designs is very popular. This method is especially used in industrial applications where the cost of experiments is expensive. Standard classical approaches are not appropriate to analyze data from saturated designs. It is due to the fact that they only allow to estimate the main factor effects and cannot assure enough freedom degrees to estimate the error variance. In this paper, we propose the use of empirical Bayesian procedures to get inferences for data obtained from saturated designs, inspired from Hadamard matrices. The proposed methodology is illustrated by assuming a dataset to prove the model robustness. The comparison between the two studied mathematical techniques, based on mean square error values (MSE), revealed that Bayesian method (BM) was more accurate than least square method (LSM): for example, the results showed that 2002 and 2000.7 mg/l for experimental and predicted (BM) data were obtained against 2002 and 1991 mg/l for experimental and predicted (LSM) data. This suggested method could be generalized in several application fields in biological sciences.

## Introduction

*Bacillus thuringiensis* (*Bt*) is a spore-forming bacterium well known for its insecticidal properties associated with its aptitude to produce crystal inclusions through sporulation. These toxins are also specific and biodegradable; therefore, no toxic products are accumulated in the environment. *Bt* biopesticides are produced by fermentation technology. The fermentation is a process in which an agent causes an organic substance to break down into simpler substances. It is considered as a major step in industrial biotechnology and it is necessary to take under consideration the optimization of medium composition to affect the formation of a specific final product during fermentation process (Schmidt 2005; Singh et al. 2008).

A fermentation enhancement program begins by measuring product yield as a response to factors like strength of medium components. Nutritional requirement can be manipulated either by the conventional or statistical approach. Conventional methods, commonly used for a long time in industrial production, involve changing one independent variable while others are kept at fixed level. However, statistical methods are more recent and offer many advantages when compared to conventional methods because they are rapid and reliable. Furthermore, these techniques are useful for short-listing significant nutrients, and then reducing the total number of experiments, hence an important gain of time and chemical products. A systematic experiment was then performed by setting the independent variables according to a statistical design carried out from Hadamard matrices at two levels and delta-endotoxins produced by *B. thuringiensis* strain was measured in each batch. A statistical analysis was then carried out to interpret the significant medium components. Such approach is a useful screening process employed to identify the contribution of each medium component to the response of the system and thus allows for a reduction of variable numbers that need to be considered (Liu et al. 2008).

In this work, we study the significance levels of medium components for delta-endotoxin production using statistical design. Moreover, we present a statistical method to identify complex ingredients interactions, during fermentation process, with Bayesian network (BN) structure learning for specific conditions. This method discovers the dependency relationship between components which implies their complex interactions on heterogeneous data sets.

## Materials and methods

### *Bacillus thuringiensis* strain

*Bacillus thuringiensis kurstaki*, an isolated strain having high delta-endotoxin production (Ennouri et al. 2013), was used in this study. The bacterial strain was grown on Luria–Bertani (LB) agar medium composed of 10 g/l of peptone, 5 g/l of NaCl, 5 g/l of yeast extract and 15 g/l of agar (BIO BASIC^®^, Ontario, Canada) and then stored at 4 °C.

### Microorganism and cultivation media

LB medium with the following composition (g/l) was used for the preparation of the pre-inoculum and inoculum: peptone, 10.0; yeast extract, 5.0; NaCl, 5.0. For fermentation medium, a modified complex medium was used (Ghribi et al. 2007). CaCO_3_ (20 g/l) was added for keeping pH stability. All media used in this study were adjusted to pH 7.0 ± 0.01 before autoclaving.

### Culture conditions

For pre-inoculum preparation, a loopful of *B. thuringiensis* grown on LB plate was used to inoculate 3 ml of sterilized LB medium and incubated in a rotary shaker (New Brunswick Scientific, model INNOVA^®^ 44, USA) at 30 °C and 200 rates per minute overnight (14–18 h). For inoculum preparation, 250-ml Erlenmeyer flasks containing 50 ml of LB medium were inoculated with 1 % (v/v) of the pre-inoculum and incubated in a rotary shaker at 30 °C and 200 rates per minute for 6 h. The volume of culture inoculum was determined on the basis of a final absorbance of approximately 0.15 measured at 600 nm. The optical density at 600 nm (OD_600_) was determined using a SmartSpec™ 3000 UV–visible spectrophotometer (Bio-Rad Laboratories). The 500-ml flasks containing 50 ml of complex medium were incubated with estimated inoculum volume. In such media, the initial OD was not measured after inoculation but calculated according to the OD measured in the inoculum. Samples taken periodically from the incubated cultures were subjected to microscopical examination. When 90 % (or more) of the *B. thuringiensis* cells had lysed, releasing the spores and crystals, the fermentation process was considered as finished.

### Determination of delta-endotoxins concentration

1 ml of collected samples at the end of fermentation was centrifuged at 13,000×*g* for 10 min at a temperature of 4 °C. The supernatants were discarded. The pellets were washed twice with 1 ml of 1 M NaCl solution and twice with 1 ml of bidistilled autoclaved water. The crystal proteins in the pellet were dissolved with 1 ml of 50 mM NaOH (pH 12.5) for 2 h at 30 °C with vigorous shaking. The suspension was centrifuged at 13,000×*g* for 10 min at 4 °C and the pellet was discarded. The supernatant containing the alkali-soluble insecticidal crystal proteins was used to define the delta-endotoxin concentration by Bradford method using bovine serum albumin as standard protein (Bradford 1976). Delta-endotoxin concentration was measured spectrophotometrically at 595 nm using a UV–visible spectrophotometer (Bio-Rad Laboratories, Inc.). The obtained values were the mean of three values of two separate experiments.

### Statistical design

Seven factors were selected as the key factors affecting the production of delta-endotoxins in this investigation. Thereafter, the screening design was applied to evaluate the importance of the seven selected factors. Plackett–Burman experimental design (Plackett and Burman 1946), an ameliorated technique based on Hadamard matrices (Hadamard 1893), was applied to evaluate the significance of various medium components affecting delta-endotoxin production by *B. thuringiensis* strain. The different factors were prepared in two levels: −1 for low level and +1 for high level, based on statistical matrix design, which is a fraction of a two-level factorial design and allows the investigation of (*n−*1) variables in at least n experiments. On the base of seven independent variables (Table 1), twelve combinations were screened according to the design shown in Table 2. All trials were performed in triplicate and the average of observations was considered as the final result. Plackett–Burman experimental design is based on the first-order model:

1 $$Y = \beta_{ 0} + \sum \beta_{\text{i}} X_{\text{i}}$$

where *Y* is the predicted response (delta-endotoxin concentration), *β*_0_ *, β*_i_ are constant coefficients, and *X*_i_ is the coded independent variable estimates or factors. The data of delta-endotoxin concentration were statistically analyzed. Factors having highest *t* value and confidence level over 95 % were considered to be highly significant on delta-endotoxin production.

**Table 1:** Coded values used in factorial design (g l^−1^)

Nutrient code	Nutrient	Minimum value (−1)	Maximum value (+1)
X1	KH_2_PO_4_	0.5	1.5
X2	K_2_HPO_4_	0.5	1.5
X3	MgSO_4_	0.1	0.5
X4	MnSO_4_	0	0.02
X5	FeSO_4_	0	0.02
X6	Starch	25	35
X7	Soybean meal	20	30

**Table 2:** Hadamard matrix of seven variables

Trial	X1	X2	X3	X4	X5	X6	X7
1	+1	+1	−1	+1	−1	−1	−1
2	−1	+1	+1	+1	−1	+1	+1
3	−1	−1	+1	+1	+1	−1	+1
4	+1	+1	−1	+1	+1	−1	+1
5	+1	−1	+1	+1	−1	+1	−1
6	−1	−1	−1	−1	−1	−1	−1
7	+1	+1	+1	−1	+1	+1	−1
8	+1	−1	+1	−1	−1	−1	+1
9	+1	−1	−1	−1	+1	+1	+1
10	−1	+1	+1	−1	+1	−1	−1
11	−1	−1	−1	+1	+1	+1	−1
12	−1	+1	−1	−1	−1	+1	+1

This method utilizes least square estimation to approximate the main effects; however, there are no degrees of freedom to estimate the error. It is difficult to obtain inferences from data of a saturated design as Plackett–Burman design; in this case, the usual analysis of variance (ANOVA) cannot be used.

Assuming that the matrix *X′X* is nonsingular in the saturated design, the least squares estimates or maximum likelihood estimation of the main effects are given as follows:

2 $$\widetilde{\beta } = (X^{\prime}X)^{ - 1} X^{\prime}y$$

In practice, generally the experimenter exploits normal plots to determine the importance of the factors; nevertheless, the interpretation of the normal plots depends on how strongly the researcher believes in factor sparsity (Baba et al. 2013). In our study, the Bayesian estimation can be applied to analyzing more deeply experimental data from saturated two-level designs.

### Bayesian estimation

We assume a Bayesian approach to analyze data from a saturated design. In the maximum likelihood approach, it is assumed that there is enough information to have a meaningful estimation of the parameters *β* and the variance *σ* ^*2*^. However, in the Bayesian approach, the data are added with information in a prior probability distribution form. The prior belief about the parameters is combined with the data’s likelihood function according to Bayes formula to give the posterior distribution of the parameters *β* and *σ* ^*2*^. Different priors could be considered to analyze data from saturated design (Baba and Gilmour 2006), but since data from saturated designs provide only limited information, the interpretation of these data depends heavily on the prior assumptions. The use of priors is very inflexible since very informative priors can lead to explicit posterior distribution (Baba and Gilmour 2006).

### Bootstrap method

The bootstrap is a well-known resampling method for evaluating the standard error of a statistical estimator, thus providing confidence intervals (Efron and Tibshirani 1993). Bootstrapping could be considered as a complementary method for model comparison and applied to further investigate the stability of the model ranking and parameter estimation. The bootstrap is a commonly used resampling method that permits estimation of the sampling variability of estimated parameters (Austin and Small 2014). Here, we used bootstrapping to test the robustness of our estimates. In fact, the bootstrap technique helps determine whether available data are sufficient for a robust classification (Wang et al. 2007). In our study, 100 simulated samples of data are used for validation: random samples were generated with replacement from a data set where each observation is randomly selected from the original data set.

## Results and discussion

### Statistical design

Seven different factors including medium components were screened for their effect on delta-endotoxin production using the experimental statistical design. The independent variables examined and their settings are shown in Table 1. The design plan is shown in Table 2. The variables *X*_1_−*X*_7_ represented the medium constituents. This method is based upon the existence of Hadamard matrices, which are square matrices of order *N* with entries at two levels, +1 and −1. These matrices are orthogonal such that for each column the number of +1 is equal to the number of −1.

**Fig. 1:**
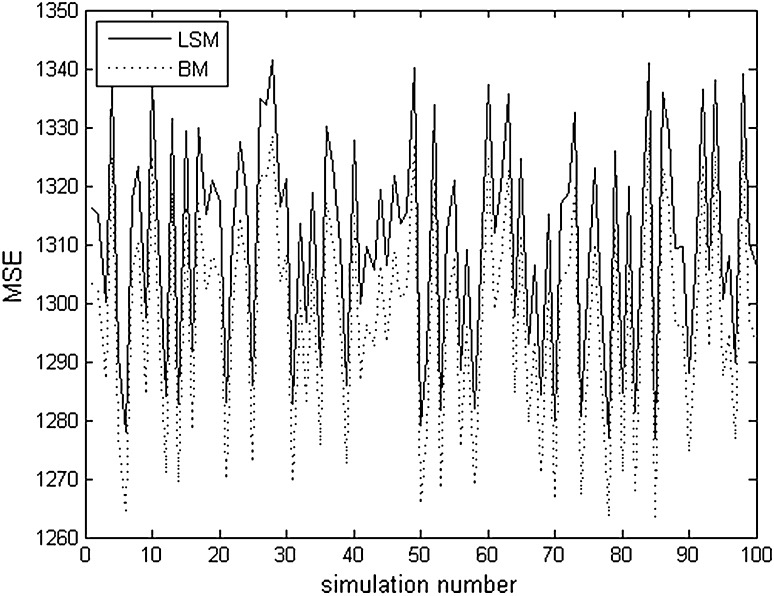
MSE variations using least square and Bayesian methods

Table 3 shows the observed and predicted concentrations of delta-endotoxins. The concentrations ranged from 1502 to 3571 mg l^−1^. The collected output data are generated from the experimental process using bootstrapping method (Efron 1979). The bootstrap is a type of Monte Carlo method applied based on observed data (Mooney and Duval 1993). The predictive distribution is presented in terms of the difference between the predicted and the observed data (Table 3). One hundred simulated samples of data are used for validation. The stopping criterion for training is a minimum value of the mean square error (MSE). In this study, the minimum value of MSE was taken as 10^−6^. The MSE was used as the criterion for the training and test data sets to compare the accuracy of the model. The enlarged versions of the simulation output based on Bayesian and linear models are presented in Fig. 1 to illustrate the difference between the mentioned variables. According to the figure, we showed that the majority of the peak points on the Bayesian method curve may indicate that this technique certainly increases goodness of model when compared to least squares method curve, and thus may improve the prediction rate. Differences in residuals between the two models are small when compared pairwise. However, the fact is that Bayesian model residuals are consistently smaller than the linear model residuals. It indicates the degree of robustness of Bayesian method compared to conventional data analysis methods [2002 and 2000.7 mg/l for experimental and predicted (LSM) data vs 2002 and 1991 mg/l for experimental and predicted (BM) data]. In fact, usual approaches such as least square method begin by fitting a model and then optimizing the model to obtain optimal operating settings. These methods do not account for any uncertainty in the parameters or in the form of the model. Bayesian approaches have been proposed recently to account for the uncertainty on the parameters of the model, assuming the model form is identified.

**Table 3:** Experimental and predicted values (least square and Bayesian methods) for the delta-endotoxin concentrations

No.	Delta-endotoxin concentration (mg l^−1^)		
	Experimental values	Predicted values (LSM)	Predicted values (BM)
1	1932	1853.66	1863.40
2	3400	3507.66	3492.80
3	2984	2759.16	2756.07
4	2562	2640.33	2637.24
5	2408	2272.5	2277.08
6	1967	1928.16	1936.41
7	2002	1991	2000.70
8	2980	3232.66	3221.47
9	3121	3014.83	3005.09
10	1680	1691	1705.85
11	1502	1754.66	1765.85
12	3571	3463.33	3446.98

In this study, a statistical design based on Hadamard matrices was employed to evaluate the main effect of the medium components for the delta-endotoxins production by *B. thuringiensis*. Table 4 provides the results of effect of each medium component on delta-endotoxins production as well as the two coefficients: *t* value and *p* value. In fact, the *t* test compares the actual difference between two means in relation to data variation, while the *p* value represents the probability that random chance can explain the end result. In general, a *p* value of 5 % or lower is considered to be statistically significant. The main effect of each variable was calculated simply as the difference between the average of measurements made at the high setting (+1) and the average of measurements observed at low setting (−1) of that factor. The components were screened at the confidence level of 95 % on the basis of their effects. That component, which showed significance at or above 95 % confidence level and its effect was positive, was interpreted as being required in higher concentration than the indicated high value (+). However, if its effect was found negative, then it indicated that the component was effective in delta-endotoxin production but the amount required was lower than the indicated low (−) concentration in Hadamard matrix. All factors in this study have shown influence on the delta-endotoxin production with confidence level at or above 95 % confidence limit and were considered to be significant for delta-endotoxins production by *B. thuringiensis* (Table 4).

**Table 4:** Regression analysis of least square method (LSM) and Bayesian method (BM)

	LSM analysis			Bayesian analysis		
Term	Coefficient	*t* value	*p* value	Coefficient	*t* value	*p* value
Constant	2509.08	35.11	0.000	2509.08	35.018	3.97e−06
X1	−8.25	−0.12	0.914	−8.25	−0.115	0.914
X2	15.41	0.22	0.840	15.42	0.215	0.840
X3	66.58	0.93	0.404	66.58	0.929	0.405
X4	−44.41	−0.62	0.568	−43.67	−0.615	0.572
X5	−200.58	−2.81	0.048	−197.28	−2.78	0.050
X6	158.25	2.21	0.091	155.67	2.19	0.093
X7	593.91	8.31	0.001	584.19	8.22	0.0012

Based on the obtained results of Table 4, the soybean meal, known as main source of organic nitrogen, showed the maximum positive effect on toxins production, followed by starch, K_2_HPO_4_ and KH_2_PO_4_. The *t* values of FeSO_4_, MnSO_4_ and KH_2_PO_4_ were negative which suggested that these components are required in the medium for delta-endotoxin production but in lower concentration than the low level. From the experimental design results, soybean meal was found to be the most significant medium component effect on delta-endotoxin production, followed by FeSO_4_. However, all other medium ingredients were found to have no significant effect on delta-endotoxin production (*p* value >0.05). Regression analysis of Bayesian method was more accurate than least square method. Therefore, the use of the proposed empirical Bayesian method in this present work could be a powerful technique and applied as complementary and profound assessment of saturated two-level designs.

The saturated factorial designs have been extensively used by industrial researchers and engineers as a powerful methodology for screening factors, especially in the presence of a great number of factors. Usually, the use of linear model based on specific experimental design in the interpretation of factorial two-level experiments could be very subjective and in many cases finding the active factors on the response of interest was quite difficult. The use of the empirical Bayesian approach introduced in this paper could be of great interest in applications.

## Conclusion

In this study, the comparison between two statistical techniques, based on mean square error values (MSE), demonstrated that the Bayesian method (BM) was more precise than least square method (LSM). The obtained results confirmed this finding and showed that the Bayesian method MSE values were smaller than least square method MSE values. For instance, according to the first run, we illustrated that concentrations of the predicted data were 1863.4 mg/l and 1853.66 mg/l using BM and LSM, respectively. Likewise, we observed excellent parameter forecasts and inferences accomplished with this suggested technique. Moreover, the Bayesian approach demonstrated the potential enhancement when spatial variability was explained in the model. This proposed method could be applied in various domains, particularly to understand complex interactions on heterogeneous data.
